# A Drifter-Based Self-Powered Piezoelectric Sensor for Ocean Wave Measurements

**DOI:** 10.3390/s22135050

**Published:** 2022-07-05

**Authors:** Seyyed Masoud Kargar, Guangbo Hao

**Affiliations:** School of Engineering and Architecture, University College Cork, T12K8AF Cork, Ireland; 120222847@umail.ucc.ie

**Keywords:** piezoelectric, sensor, ocean, sea, wave, measurement, drifter, energy, harvester

## Abstract

Recently, piezoelectric materials have received remarkable attention in marine applications for energy harvesting from the ocean, which is a harsh environment with powerful and impactful waves and currents. However, to the best of the authors’ knowledge, although there are various designs of piezoelectric energy harvesters for marine applications, piezoelectric materials have not been employed for sensory and measurement applications in marine environment. In the present research, a drifter-based piezoelectric sensor is proposed to measure ocean waves’ height and period. To analyze the motion principle and the working performance of the proposed drifter-based piezoelectric sensor, a dynamic model was developed. The developed dynamic model investigated the system’s response to an input of ocean waves and provides design insights into the geometrical and material parameters. Next, finite element analysis (FEA) simulations using the commercial software COMSOL-Multiphysics were carried out with the help of a coupled physics analysis of Solid Mechanics and Electrostatics Modules to achieve the output voltages. An experimental prototype was fabricated and tested to validate the results of the dynamic model and the FEA simulation. A slider-crank mechanism was used to mimic ocean waves throughout the experiment, and the results showed a close match between the proposed dynamic modeling, FEA simulations, and experimental testing. In the end, a short discussion is devoted to interpreting the output results, comparing the results of the simulations with those of the experimental testing, sensor’s resolution, and the self-powering functionality of the proposed drifter-based piezoelectric sensor.

## 1. Introduction

After the introduction of piezoelectricity [1], piezoelectric materials have gained considerable attention in research and industry over time. In response to applied stress, piezoelectric materials change their atomic configuration to form dipoles, in which electrical pulses are generated. This working state is called the direct piezoelectric effect, which produces an alternating current (AC) voltage output as a result of the applied periodic force. Conversely, these materials, under the application of an electrical voltage, extend or contract, which is called the converse piezoelectric effect. Employing these two phenomena, direct and converse piezoelectric effects, piezoelectric materials are utilized in various applications as sensors, actuators, and energy harvesters [2].

Taking advantage of the direct piezoelectric effect, different piezoelectric-based sensors have been designed for measuring pressure and stress [3,4,5], acceleration [6,7,8], and tactile stimuli [9,10], medical diagnostics [11,12], acoustics [13], structural health monitoring [14], etc. Moreover, using the same principle, many energy harvesters have been designed based on the piezoelectric principle for different applications, e.g., in transportation [15], wearable electronics [16], microfluidics [17], wind and ocean energies [18,19], and biomedical engineering [20].

In 1970, piezoelectric materials started to be utilized in oceanic applications for energy harvesting purposes [2]. The energy harvested by these materials from the oceans can be used for powering low-power devices such as sensors, the internet of underwater things, etc., and make them battery-free [21]. Compared to their counterparts in generating small-scale electricity such as electrostatics [22], electromagnetics [23], and triboelectric [24] devices, piezoelectric energy harvesters have a higher energy generation density [25]. Moreover, they do not require frequent maintenance and can be simply attached to oceanic devices to support their power [26]. Based on their configurations, piezoelectric energy harvesters can be classified into four categories: (1) cantilever beam configuration; (2) diaphragm configuration; (3) cymbal configuration; (4) stacked configuration [27].

In our former work [27], we reviewed all the piezoelectric energy harvesters in oceanic applications and made an atlas for further analysis. The atlas includes all the information about the designs’ material, coupling modes, location, power density, power source, and schematics of their structure. However, to the best of our knowledge, although there are various designs of oceanic piezoelectric energy harvesters, there is no single design of piezoelectric sensors for oceanic measurements.

Being harsh, the marine environment requires regular and real-time measurements. There are many facilities and sites throughout the oceans, and it is of high significance to prevent any possible damage to them due to the ocean’s powerful and impactful waves. Therefore, sensors are required to measure oceanic waves to predict their power and reduce the risk of damage to oceanic facilities and sites [28].

**Figure 1:**
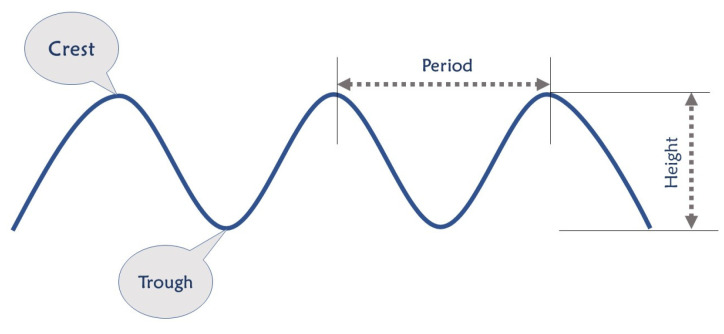
Ocean wave characteristics [27].

The association of atmospheric pressure gradients, gravitational attraction, earthquakes, storms, and wind forces would result in the generation of ocean waves [29]. As shown in Figure 1, there are two main features of an oceanic wave that can be used to determine its power: the height and the period. A wave having a greater height with a shorter period contains more power [30]. Hence, developing novel methods is required to measure these two important features of a wave in real time for damage prevention in oceanic sites. Currently, oceanic waves are monitored by several techniques such as buoys, micro-seism analysis, coastal radars, and satellite observations. Each of the techniques has its limitations. For example, having a long anchor in buoys causes problems for fishing in the oceans. Moreover, these systems contain sensitive devices that can be easily damaged. In the case of radar sensors, they show a low signal-to-noise ratio, which limits the accuracy of detecting low wave heights. Satellite sensors also show imperfect radiometric, spatial, and spectral resolutions [31].

In the present research, using the same principle as in piezoelectric energy harvesters, a drifter-based piezoelectric sensor is proposed to measure oceanic waves’ height and period, which has the following advantages:

- Providing real-time measurements;
- Accurately measuring oceanic waves, even those whose magnitude is very small;
- Precisely measuring the waves’ period;
- Having a simple but robust structure;
- Capable of being self-powered;

This paper is organized as follows: Section 2 presents the design and working principles of the proposed drifter-based piezoelectric sensor; in Section 3, a dynamic model based on the well-known spring–mass–damper system is proposed to analyze the motion principle and the working performance of the sensor; in Section 4, an FEA simulation using COMSOL Multiphysics is performed to verify the dynamic model’s results; in Section 5, prototyping and experimental testing setups are shown; in Section 6, interpretation of the output voltage and comparison of the results, resolution of the sensor, and sensor’s self-powering capability are carried out; in the end, in Section 7, conclusions are drawn, and future work is discussed.

## 2. Drifter-Based Piezoelectric Sensor

**Figure 2:**
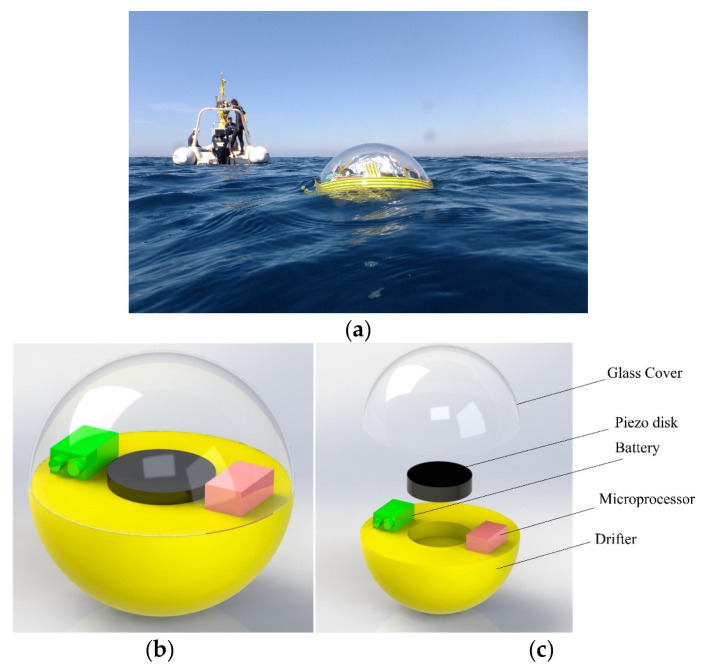
(**a**) An example of oceanic drifters by Carandell et al. [33] (**b**) Proposed drifter-based self-powered piezoelectric sensor for ocean wave measurement; (**c**) Sensor’s components (Glass cover, piezo disk, battery, microprocessor, and drifter).

**Figure 3:**
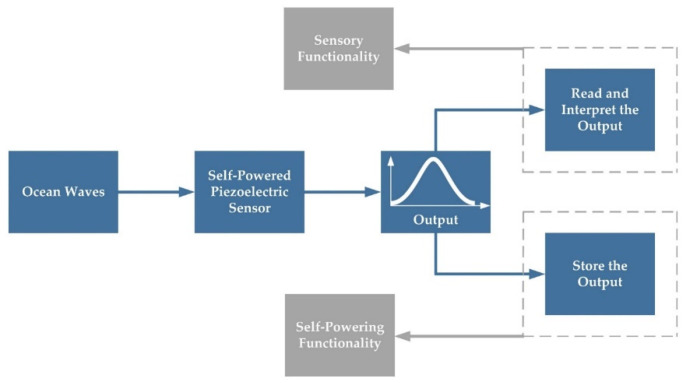
Working principle of the self-powered piezoelectric sensor.

Oceanic drifters are floating devices that carry different measurement instruments for physical and biological oceanography (Figure 2a). Transported by ocean currents and waves, drifters are also used to map flow patterns [32]. Here, a drifter was employed to carry the sensor and help it float on the ocean surface. Figure 2b shows a schematic of the proposed drifter-based piezoelectric sensor. It consists of a drifter, a piezoelectric disk, a battery, a microprocessor, and a glass cover (Figure 2c). As discussed in Section 1, piezoelectric materials generate electricity in response to mechanical stresses. Therefore, the output voltage can be used to capture ocean waves’ height and period. Moreover, this electrical energy can be stored in a battery and further used to power the sensor and send the captured data to a nearby station (Figure 3). The glass cover is also used to seal the electronics protecting it from ocean water.

The proposed drifter-based piezoelectric sensor works based on Newton’s first law of motion (the law of inertia), in which an object resists any changes in its state of motion due to its mass. Ocean waves start to fluctuate and apply a force to the drifter and, consequently, to the piezoelectric disk, thus tending to change their state of motion. Because of inertia, the drifter and the piezoelectric disk resist to changing their state of motion; thus, the piezoelectric disk deforms due to this force and generates electricity in response to it. The generated electrical pulses can be used to power the sensor (charge the battery) and capture waves’ height and period. After powering the battery, the sensor can send the output data to a nearby station for further analysis.

**Figure 4:**
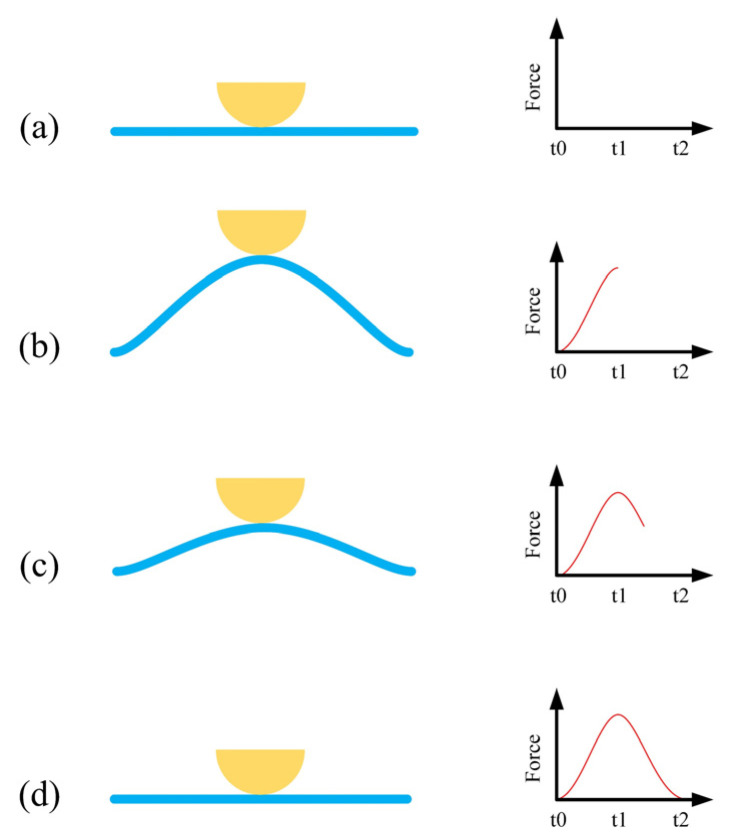
Working steps of the drifter-based piezoelectric sensor: (**a**) At time t_0_; (**b**) From t_0_ to t_1_; (**c**) From time t_1_ to t_2_; (**d**) At time t_2_.

The working steps of the proposed drifter-based piezoelectric sensor are as follows (Figure 4):

- **At time t_0_:** The ocean waves are at rest, and the forces are in balance (Figure 4a);
- **From time t_0_ to t_1_:** Ocean waves increase and apply a force to the drifter and the piezoelectric disk, tending to change their state of motion. Resistance to this change would result in the deformation of the piezoelectric disk and electricity generation (Figure 4b);
- **From time t_1_ to t_2_:** After a while, the ocean waves gradually subside, resulting in a reduction in the forces on the piezoelectric disk (Figure 4c);
- **At time t_2_:** The ocean waves are at rest again, and the forces are in balance (Figure 4d);

## 3. Dynamic Modeling

**Figure 5:**
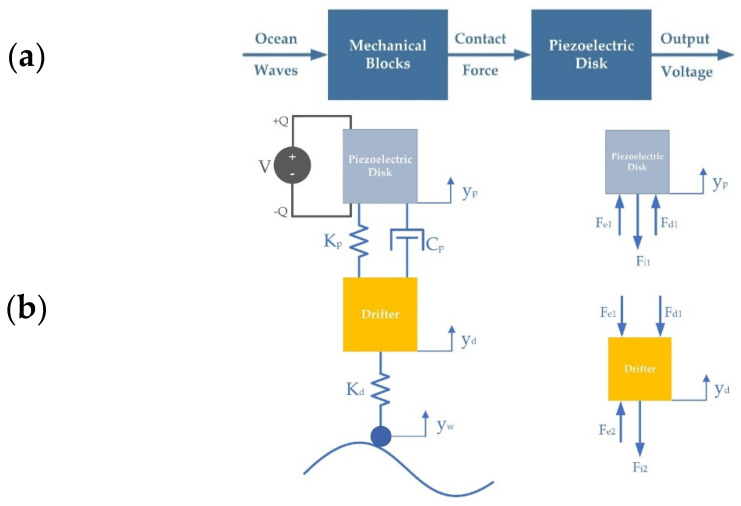
(**a**) Steps of the dynamic modelling from ocean waves to output voltages; (**b**) Schematics of the proposed dynamic model and its free-body diagram.

Based on Newton’s second law of motion, a dynamic model was established to analyze the motion principle and the working performance of the proposed drifter-based piezoelectric sensor. The main purpose of this modeling was to analyze the system’s response (the output) to an input of ocean waves. In the first step, the contact force on the piezoelectric material was calculated and then it was substituted into the related equations to obtain the output voltages. Figure 5a shows the steps of the dynamic modelling from ocean waves to the output voltages. 

Schematics of a dynamic model and its free-body diagram are illustrated in Figure 5. A damped spring–mass system was utilized to investigate the dynamic performance of the proposed sensor. The mass, stiffness, displacement, and damping coefficient of the piezoelectric disk are represented by *M*_p_, *K*_p_, *Y*_p_, and *C*_p_, respectively. Moreover, the mass, stiffness, and displacement of the drifter are *M*_d_, *K*_d_, and *Y*_d_, respectively. *Y_w_* denotes the motion of the ocean waves.

In Figure 5:

F_i1_ = piezoelectric disk’s inertial force;
F_i2_ = drifter’s inertial force;
F_e1_ = piezoelectric disk’s elastic force;
F_e2_ = drifter’s elastic force;
F_d1_ = piezoelectric disk’s damping force.

The direction of the forces was determined by considering that the ocean waves have a motion like that of a sine wave, which causes the sensor to move accordingly. The force acting on the drifter from the waves will cause the drifter to compress. This will generate an elastic force, *F_e_*_2_, which will push the drifter up. Due to inertia, the drifter will try to resist the position change through the inertial force *F_i_*_2_. The elastic force also acts on the ocean waves, but this was not of interest in this study. The piezoelectric disk will oppose the upward movement of the drifter and will push on it with the elastic force *F_e_*_1_ and the damping force *F_d_*_1_. According to Newton’s third law of motion, for each action, there is a reaction. Therefore, the elastic force *F_e_*_1_ and the damping force *F_d_*_1_ will push the piezoelectric disk upwards. Due to inertia, the body’s mass will try to resist the position change through the inertial force *F_i_*_1_.

The equation of force equilibrium for the piezoelectric disk is:

*F_i_*_1_ = *F_e_*_1_ + *F_d_*_1_, (1)

The inertial force and the elastic and damping forces of the piezoelectric disk are expressed as:

(2) $${F_{i}}_{1}\;=\;M_{p}{\ddot{y}}_{p},$$

(3) $${F_{d}}_{1}\;=\;C_{p}\left({\dot{y}}_{c}-{\dot{y}}_{p}\right),$$

(4) $${F_{e}}_{1}\;=\;K_{p}\left(y_{c}-y_{p}\right),$$

Replacing the expressions of the forces in Equation (1), we obtain:

(5) $$M_{p}{\ddot{y}}_{p}=C_{p}\left({\dot{y}}_{d}-{\dot{y}}_{p}\right)+K_{p}\left(y_{d}-y_{p}\right),$$

The equation of force equilibrium for the drifter is:

*F_i_*_2_ + *F_e_*_1_ + *F_d_*_1_ = * F_e_*_2_, (6)

The inertial force and the elastic and damping forces of the drifter are expressed as:

(7) $${F_{i}}_{2}\;=\;M_{d}{\ddot{y}}_{d},$$

(8) $${F_{e}}_{2}\;=\;K_{d}\left(y_{w}-y_{d}\right),$$

Substituting the expressions of the forces in Equation (6), we obtain:

(9) $$M_{d}{\ddot{y}}_{d}+C_{p}\left({\dot{y}}_{d}-{\dot{y}}_{p}\right)+K_{p}\left(y_{d}-y_{p}\right)=K_{d}\left(y_{w}-y_{d}\right),$$

Applying the contact force (*F_e_*_1_ *=* $K_{p}\left(y_{c}-y_{p}\right)$ and *F_d_*_1_ *=* $C_{p}\left({\dot{y}}_{c}-{\dot{y}}_{p}\right)$) on the piezoelectric disk by introducing it in the following equations (Equations (10)–(12)) [34] will give us the output voltage generated:

(10) $$V=\frac{Q}{C}\;,$$

where *V*, *Q*, and *C* are voltage, charge, and capacitance of the piezoelectric disk, respectively. Q and C can be simplified using the following equations [35]:

*Q* = *d*_33_ × *F*, (11)

(12) $$C=\frac{\varepsilon_{r}\varepsilon_{0}A}{t}\;,$$

where *d*_33_, *F*, $\varepsilon_{r}$, $\varepsilon_{0}$, *A*, and *t* represent piezoelectric coefficient, contact force, relative permittivity, vacuum permittivity, area, and thickness, respectively.

Moreover, from the following equations, mass and stiffness for both the drifter and the piezoelectric disk can be calculated, and the damping and piezoelectric coefficients of the piezoelectric disk can be taken from references:

(13) $$\rho =\frac{M}{v}\;,$$

(14) $$K=E\frac{A}{l}\;,$$

where ρ and *v* are the density and volume of the material, and E, A, and l stand for Young’s modulus, area, and length of the material, respectively.

Taking advantage of the MATLAB/Simulink modulus, the aforementioned proposed dynamic model was simulated. Table 1 shows the parameters used in the simulation. Some of them were taken from the literature, while others are based on the prototype and FEA model. It should also be mentioned that Polyvinyl Chloride (PVC) was employed for the drifter in the simulations.

**Figure 6:**
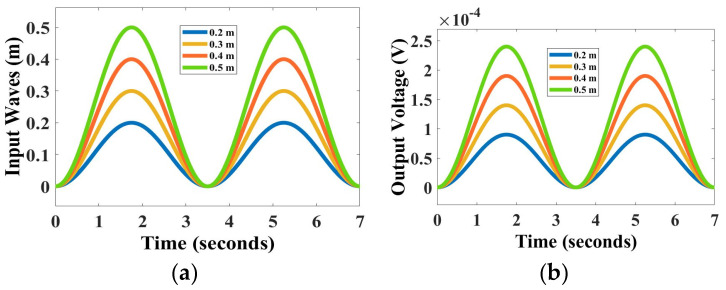
Input waves and output voltages: (**a**) Input ocean waves of height from 0.2 to 0.5 m; (**b**) Output voltages for ocean waves of 0.2 m (Blue line), 0.3 m (Orange line), 0.4 m (Red line), and 0.5 m (Green line) height.

Figure 6 shows the input waves to the developed dynamic model (Figure 6a) and the resulting output voltages (Figure 6b) for ocean waves with a height from 0.2 to 0.5 m and a 3.5 s period.

The peak values of the output voltages were 90 µV, 140 µV, 190 µV, and 240 µV for input waves with 0.2 m, 0.3 m, 0.4 m, and 0.5 m height, respectively. A 3.5 s period was chosen for all input waves in the dynamic model.

## 4. FEA Simulations

**Figure 7:**
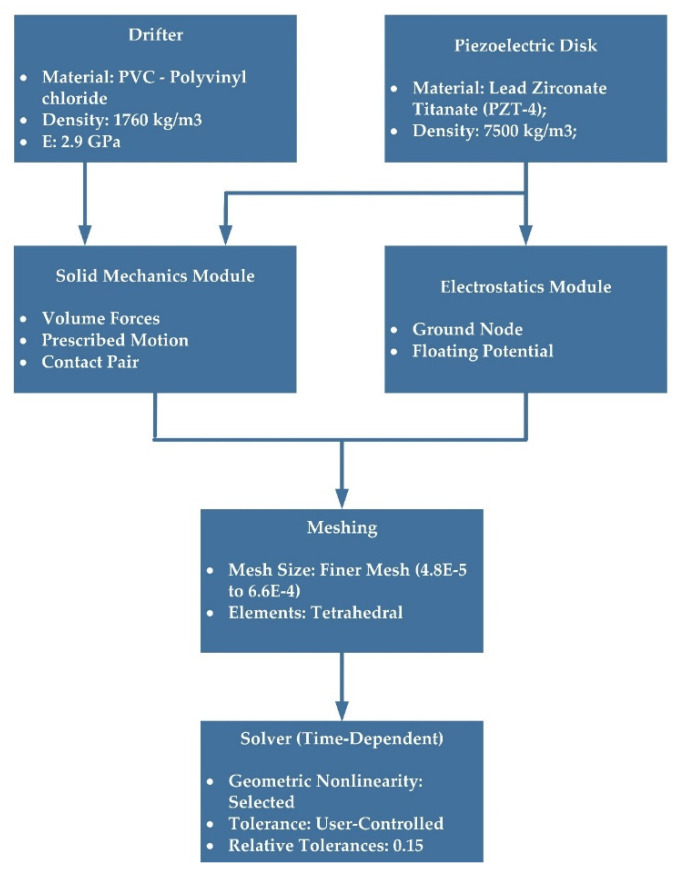
Flowchart of the FEA simulation in COMSOL.

**Figure 8:**
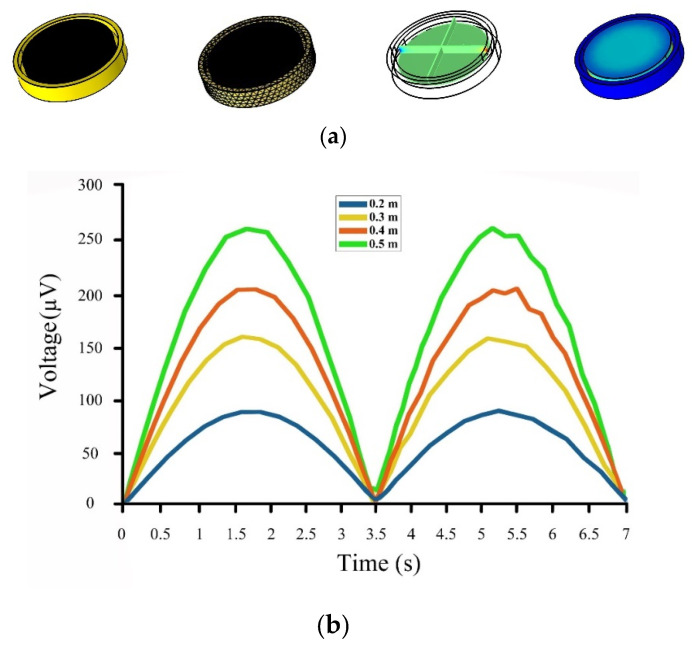
COMSOL models and results: (**a**) Model, meshed model, electrical potential output, and stress output; (**b**) Output voltages for ocean waves of 0.2 m (Blue line), 0.3 m (Yellow line), 0.4 m (Red line), 0.5 m (Green line) height.

An FEA simulation using the commercial software COMSOL-Multiphysics was carried out to further verify the feasibility of the designed drifter-based piezoelectric sensor. To do so, coupled physics analyses of “Solid Mechanics” and “Electrostatics” Modules were used to simulate the piezoelectric sensor and determine the output voltages. For this analysis, Lead Zirconate Titanate (PZT-4) and Polyvinyl Chloride (PVC) were used for the piezoelectric material and the drifter, respectively, and a “Finer” element size was employed for meshing the structure. A Ground node was applied on the lower side of the piezoelectric material, and a Floating Potential node was applied on its upper side. A contact pair was utilized to define the contact between the piezoelectric disk and the drifter. Then, volume forces (gravity) were applied to both of them. Finally, a prescribed motion with a sinusoidal function was applied to the drifter to mimic the ocean waves, which was the input of the system. Details of the steps of the FEA model can be found in Figure 7. The sensor, meshed model, and results of the simulations are shown in Figure 8.

As can be seen in Figure 8, the results of the FEA simulations for the proposed drifter-based piezoelectric sensor in the commercial software COMSOL Multiphysics were a bit higher than those of the proposed dynamic model. The peak values for the output voltage were 95 µV, 160 µV, 205 µV, and 260 µV for input ocean waves of 0.2 m, 0.3 m, 0.4 m, and 0.5 m height, respectively. It should also be noted that the period of the input wave for all of the above-mentioned simulations was 3.5 s.

## 5. Prototype Fabrication and Testing

**Figure 9:**
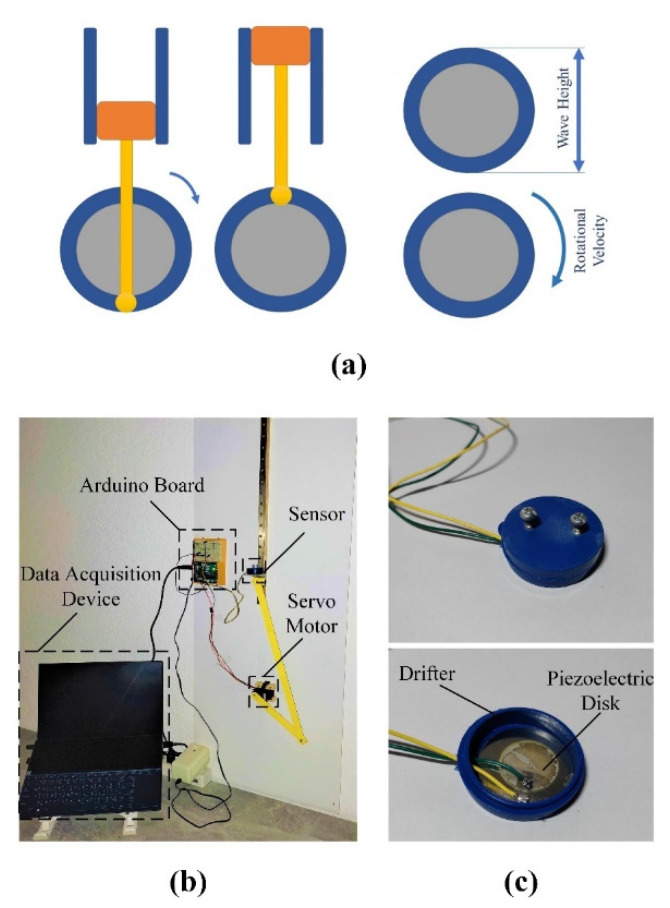
Experimental setup and results: (**a**) Slider-crank mechanism; (**b**) Experimental setup; (**c**) The fabricated sensor prototype for testing.

**Figure 10:**
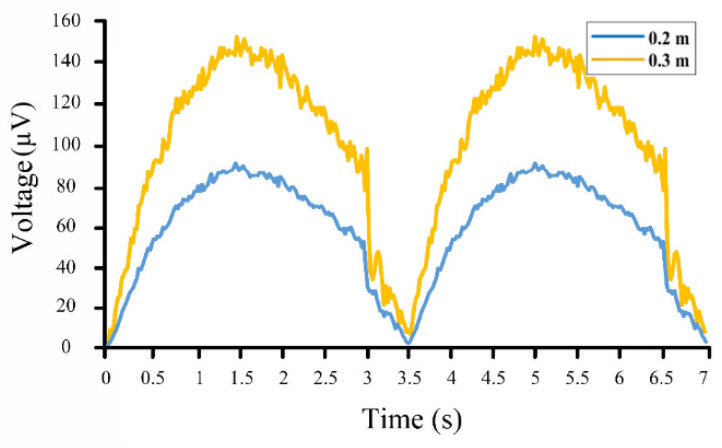
Results of the fabricated prototype for waves of 0.2 m (Blue line) and 0.3 m (Orange line) height with a 3.5 s period.

To simulate the motion of the ocean waves, a slider-crank mechanism was employed, in which the circular velocity of the motor represents the ocean waves’ period, and its diameter stands for the waves’ height (Figure 9a). Simply speaking, the diameter of the disk specifies distance between a wave’s crest and trough, and its rotational velocity determines the wave’s period (frequency). As shown in Figure 9b, the experimental setup included a slider-crank mechanism, a servo motor, the proposed sensor, an Arduino board, and a Personal Computer (PC) as the data acquisition device. The fabricated sensor is shown in Figure 9c, in which a piezoelectric disk (a buzzer element STD 35 mm manufactured by Murata Electronics) was nested inside a plastic container that represented the drifter shown in Figure 2. The programs for the servo motor’s motion were set on the PC and then uploaded to the Arduino board. Taking orders from the board, the servo motor started working with the determined rotational velocity. Then, the slider-crank mechanism represented the ocean waves and pushed the piezoelectric sensor up. The output voltage of the piezoelectric sensor was recorded by the Arduino board and sent to the PC, which is shown in Figure 10. This Figure shows the results of the simulation for ocean waves with a period of 3.5 s and heights of 0.2 m and 0.3 m. It should be mentioned that the experiment was limited to these two waves (heights of 0.2 m and 0.3 m and a period of 3.5 s) due to the employed servo motor and the experimental setup. 

The output results of the experimental setup were mainly affected by the coarse motion of the slider-crank mechanism. As it is clear from Figure 10, the slider-crank mechanism carried the sensor up with a bit of noise. However, due to the action of gravity, it came down very fast. Fortunately, the proposed sensor could successfully plot these results as well.

## 6. Discussion

### 6.1. Interpretation

**Figure 11:**
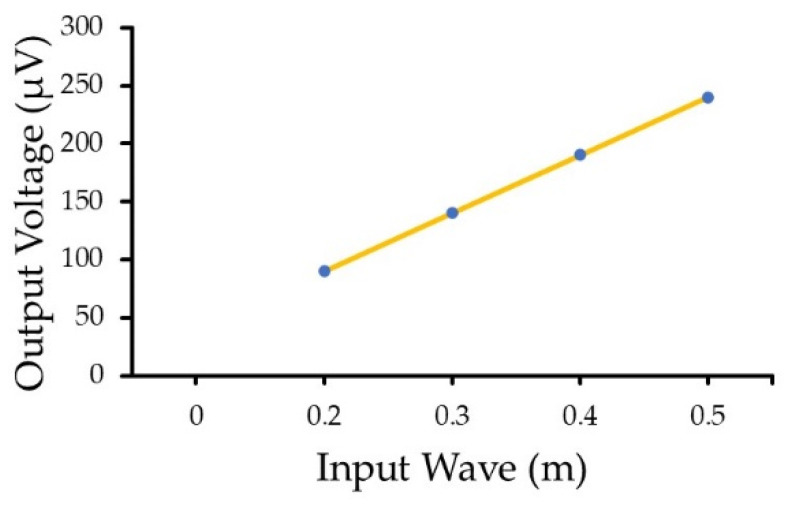
Linear relationship between the output voltage and the input ocean waves.

As discussed in Section 1, a wave’s energy can be estimated based on its height and period. Here, the proposed drifter-based piezoelectric sensor could precisely measure these two elements to finally obtain a wave’s energy. As it is clear from the dynamic model, FEA simulation, and experimental results (Figure 6, Figure 8 and Figure 10), the wave’s period is the same as the output voltage of the piezoelectric material. On the other hand, based on the results of the dynamic model, there is a linear relationship between the output voltage and the wave height for different input waves (Figure 11). Therefore, the wave height can also be calculated simply from the output voltage of the proposed piezoelectric sensor. For example, considering the results of the proposed dynamic model (Figure 6), for each 0.1 m change in the wave’s height, there would be a 50 µV change in the output voltage, meaning that by substituting the output voltage in the following equation, the wave’s height can be measured:

(15) $$H\approx \frac{V_{out}}{5\times 10^{-4}}\;,$$

where *H* and $V_{out}$ are the wave’s height and the maximum output voltage, respectively.

### 6.2. Results Comparison

**Figure 12:**
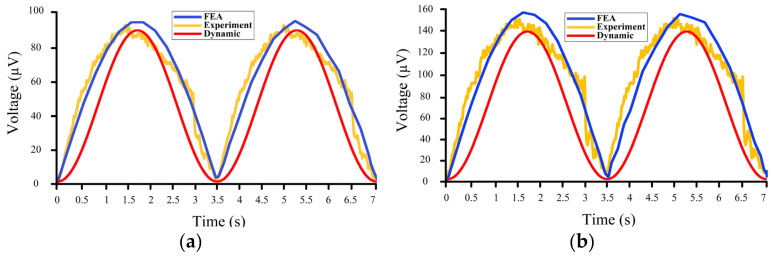
Comparison of the results of the dynamic model (Red line), FEA simulation (Blue line), and experiment (Orange line): (**a**) Results for 0.2 m wave height; and (**b**) Results for 0.3 m wave height.

The output results of the models and the experiments are plotted in Figure 12. These results are for input waves of 0.2 m (Figure 12a) and 0.3 m (Figure 12b) heights with a 3.5 s period. Moreover, Table 2 lists and compares the peak values of the output voltages in the simulations and the experiment.

**Figure 13:**
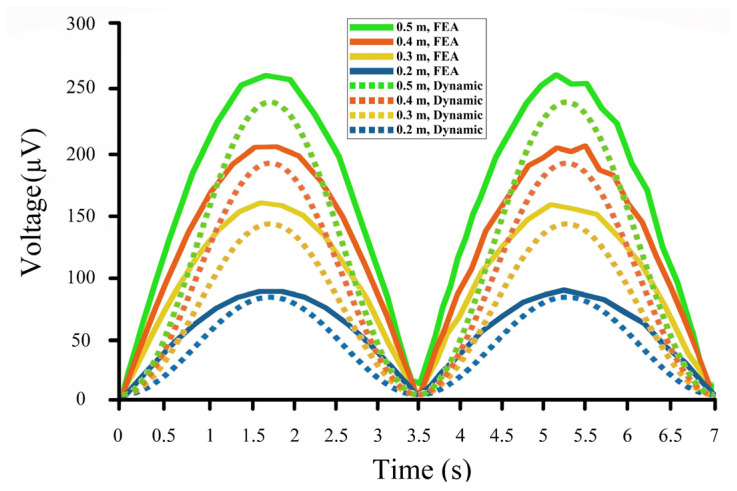
Results of the dynamic model (dashed lines) and the FEA simulation (lines).

As can be seen in Table 2, the average errors of the results are about 8%, which further verifies the feasibility of the proposed drifter-based piezoelectric sensor for oceanic wave measurements. There are also noises in the results of the experimental testing compared to those of the other two models, which were mainly caused by the coarse motion of the slider-crank mechanism. Moreover, to further compare the results of the dynamic model and the FEA simulation, the outputs were plotted in Figure 13.

### 6.3. Resolution

**Figure 14:**
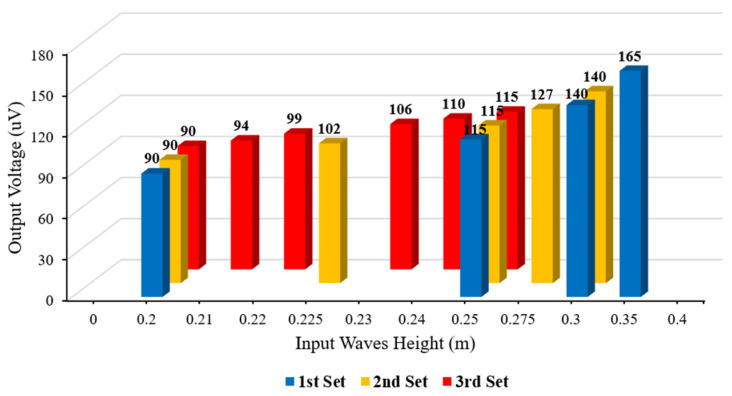
Resolution testing of the sensor based on the proposed dynamic model.

A set of analyses based on the proposed dynamic model in Section 3 was designed to determine the resolution of the proposed sensor. To do so, three types of input waves were applied to the sensor to see whether it could distinguish them. The input waves had a difference of 0.05 m, 0.025 m, and 0.01 m in each set of inputs: (1) 0.2 m, 0.25 m, 0.3 m, and 0.35 m for the first set; (2) 0.2 m, 0.225 m, 0.25 m, 0.275 m, and 0.3 m for the second set; and (3) 0.2 m, 0.21 m, 0.22 m, 0.23 m, 0.24 m, and 0.25 m for the third set. Based on the output voltages for the designed sets of input waves, the sensor could successfully distinguish input waves with a difference of 0.05 m and 0.025 m in height, and a linear relationship is clear in Figure 14. However, the output voltages for input waves with a 0.01 m difference were not linear, thus the proposed sensor can only measure ocean waves that differ more than 0.025 m in height.

### 6.4. Self-Powering

As discussed in Section 2, the proposed piezoelectric sensor can store the generated electricity in response to the ocean waves for further use to send the extracted data to a nearby station. In this regard, the sensor itself can be considered a piezoelectric energy harvester, which has been a favored subject of research in recent oceanic research [27]. In the case of the present sensor, as the generation of the output voltage was demonstrated in Section 3, Section 4 and Section 5 using dynamic modeling, FEA simulation, and experimental testing, the output power that can be stored in a battery would be [36]:

(16) $$P=\frac{1}{T}\int_{0}^{T}\frac{V^{2}}{R}dt,$$

where *P*, *R*, *V*, and *T* are power, resistance, voltage, and period of *V*, respectively. It should be noted that, based on Equation (16), the generated power by the present configuration is very low (nW − µW) and should be improved to power the sensor in a real environment independently.

**Figure 15:**
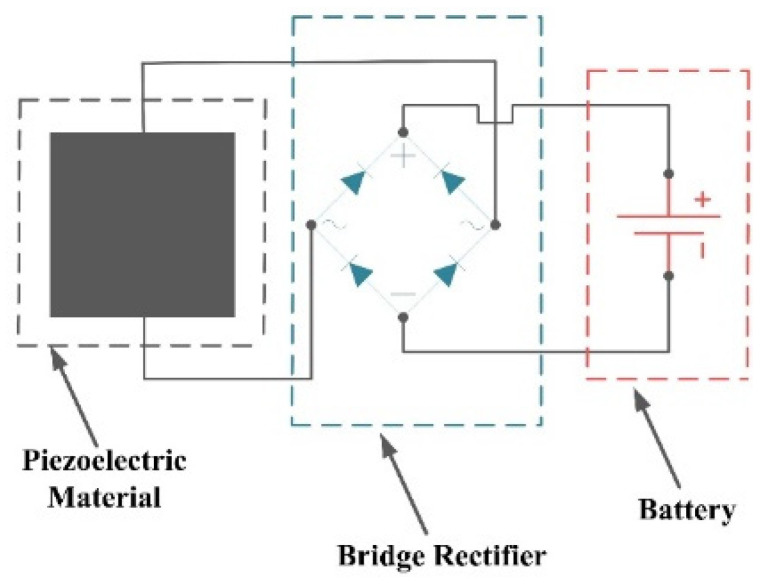
Circuit of the energy harvester to store the generated electricity in a battery.

The battery as the power storage system of the sensor requires further processing of the generated electricity by the piezoelectric material: batteries require direct current (DC), and the piezoelectric energy harvester generates alternative current (AC), which should be rectified before being stored in the battery. Therefore, adding an intermediate step to the circuit of the harvester would convert AC signals to DC signal [27]. Figure 15 shows the circuit of the energy harvester in the proposed piezoelectric sensor.

## 7. Conclusions

In this research, to measure ocean waves’ height and period, a novel drifter-based piezoelectric sensor is proposed, which is capable of being self-powered. A dynamic model was developed to analyze the proposed sensor’s working performance and motion principle. To verify the results of the dynamic model, an FEA simulation using COMSOL-Multiphysics was performed. Both Solid Mechanics and Electrostatics Modules were employed to develop a coupled-physics simulation for the proposed sensor. To validate the results of the dynamic model and the FEA simulation, a prototype was fabricated and tested, and the results showed a close match between the proposed dynamic modeling, FEA simulations, and experimental testing. The average error between the results was about 8%, which verified a close match between the results. Through analyses based on the developed dynamic model, the resolution of the sensor was calculated, and the sensor appeared suitable for measuring oceanic waves of 0.025 m in height. Finally, the sensor’s energy harvesting functionality, which allows the sensor to become self-powered, was discussed and demonstrated. This research is a preliminary validation for using the output of the piezoelectric materials for sensory applications in oceans. Several items should be addressed in our future works:

(1) Fabricating a new prototype for real-environment testing;
(2) Developing algorithms to train the sensor for autonomous purposes;
(3) The other important issue that should be addressed in future research is the interpretation of the outputs for complex output voltages. To do so, some advanced mathematics can be used to interpret the results for complex input waves that are frequent in real environments;
(4) Working on developing techniques to send the extracted data to a nearby station;
(5) Working more on the energy harvesting functionality of the sensor to enhance the output power to charge a battery and power the sensor in a real environment;
(6) Design experiments to realize the expected range of operation for the sensor: obviously, below a certain value, the noises dominate the output of the sensor, so the sensor will not be able to measure them. Moreover, higher accelerations can harm the piezoelectric material’s performance. Therefore, there is also a maximum wave height for the sensor to precisely measure.
(7) There are factors such as temperature fluctuations in oceans, maximum vibrational working cycles, etc., which influence the working performance of the piezoelectric material in long-term applications that need to be considered in future work;
(8) Improving the structure and configuration of the sensor by adding a seismic mass to the piezoelectric material to enhance its sensitivity.

## Figures and Tables

**Table 1:** Parameters used in the MATLAB/Simulink simulation.

Parameter	Value	Parameter	Value
*d*_33_	2.89 × 10^−10^ m/V	The density of Piezo	7500 kg/m^3^
$\varepsilon_{r}$	1300	The density of the Drifter	1760 kg/m^3^
$\varepsilon_{0}$	8.85 × 10^−12^	Diameter of the Drifter	40.00 × 10^−3^ m
Thickness of Piezo	1.00 × 10^−3^ m	Height of the Drifter	10.00 × 10^−3^ m
Area of Piezo	3.14 × 10^−4^ m^2^		

**Table 2:** Comparison of the results.

		0.2 m	0.3 m	0.4 m	0.5 m
**Results**	Dynamic Model	90 µV	140 µV	190 µV	240 µV
	FEA	95 µV	160 µV	205 µV	260 µV
	Experiment	92 µV	155 µV	-	-
**Error (%)**	Dynamic and FEA	5.5	14.2	7.8	8.3
	Dynamic and Experiment	2.2	10	-	-
	FEA and Experiment	3.2	3.2	-	-
